# Draft genome sequence of *Clostridium jeddahense* EE-R19 isolated from an anaerobic digester

**DOI:** 10.1186/s13104-020-05247-3

**Published:** 2020-09-03

**Authors:** Elvira E. Ziganshina, Waleed S. Mohammed, Ayrat M. Ziganshin

**Affiliations:** 1 Department of Microbiology, Institute of Fundamental Medicine and Biology, Kazan (Volga Region) Federal University, Kremlyovskaya Str. 18, Kazan, 420008 Russia; 2 Department of Biotechnology, Faculty of Agriculture, Al-Azhar University, Cairo, 11651 Egypt

**Keywords:** Draft genome, *Firmicutes*, *Clostridium jeddahense*, Anaerobic digester

## Abstract

**Objectives:**

*Clostridium* species of the order *Clostridiales* are mostly strictly anaerobic rod-shaped bacteria. They can be detected in a variety of environments, including the intestines of humans and animals, soil, water, and biogas reactors. Species of the genus *Clostridium* are widely used in various biotechnological processes, but several of them have been identified as significant human pathogens. Therefore, investigation at the genome level is necessary to provide valuable information about the ecology, genetics, and phylogenetic diversity of various *Clostridium* species.

**Data description:**

In the present study, we report the whole genome sequence of *Clostridium jeddahense* strain EE-R19, which was isolated from a mesophilic anaerobic digester. The draft genome of *C. jeddahense* EE-R19 consisted of 59 contigs (> 500 bp), which amounted to 3,562,974 with an overall G + C content of 51.79%. The whole genome shotgun project of *C. jeddahense* EE-R19 has been deposited at DDBJ/ENA/GenBank under the accession number JAAVNF000000000.

## Objective

Members of the order *Clostridiales* can be found in a variety of environments, including the intestines of humans and animals, soil, water, marine environments, and biogas reactors [1–4]. The clostridia are basically strict anaerobes with gram-positive staining and the ability to sporulate. Their metabolism varies greatly because they can metabolize various compounds, including carbohydrates, proteins, alcohols, amino acids, and purines. Organic acids and alcohols can be obtained during the metabolism of carbohydrates or proteins. However, some of them have been identified as human pathogens [1]. The genome data will expand understanding of the genus *Clostridium* and provide valuable information about ecology and genetics of the species *Clostridium jeddahense*.

### Data description

*Clostridium jeddahense* strain EE-R19 was originally isolated from anaerobically digested distillers grains and cow manure at mesophilic temperatures on nutrient agar plates in a BACTRON anaerobic chamber (Shel Lab) in Kazan, Republic of Tatarstan, Russia. The strain *C. jeddahense* EE-R19 was then cultivated on nutrient agar plates at + 38 °C for 72 h in a BACTRON anaerobic chamber. Biomass from a culture of *C. jeddahense* EE-R19 was harvested, washed twice with sterile K-Na-phosphate buffer (pH 7.0), and genomic DNA was extracted by using FastDNA spin kit (MP Biomedicals) according to the manufacture’s protocol. The identity of the strain EE-R19 was confirmed based on morphological, biochemical, and growth characteristics, and finally by sequencing its 16S rRNA gene (Table 1) (16S rRNA gene sequence, 1385 bp, BLAST identity of 99.33% to *Clostridium jeddahense* strain JCD (NR_144697.1)). In addition, the average Nucleotide Identity (ANI) values were estimated using the JSpeciesWS online service for the comparison of the whole genome sequences [5], which confirmed that the strain EE-R19 belongs to the *C. jeddahense* species.

**Table 1:** Overview of data files/data sets

Label	Name of data file/data set	File types (file extension)	Data repository and identifier (DOI or accession number)
Data set 1	*C. jeddahense* EE-R19, whole genome shotgun sequencing project	FASTA	Genbank (https://www.ncbi.nlm.nih.gov/nuccore/JAAVNF000000000.1)
Data set 2	*C. jeddahense* EE-R19, 16S ribosomal RNA gene, partial sequence	FASTA	Genbank (https://identifiers.org/ncbi/insdc:MT253587.1)

DNA libraries were created as previously reported [6, 7] and according to the Illumina protocol. The genome was then sequenced using an Illumina MiSeq system on a paired-end library with MiSeq Reagent Kit v3 (600-cycle). Quality assessment of the FASTQ sequence files was based on the FastQC software (version 0.11.8) [8]. Velvet assembler (version 1.2.10) was used to assemble reads into contigs [9]. Mauve program (version 2.4.0) was used as a contig ordering tool [10]. The bacterial genome annotation was achieved by uploading the genome assembly of the *C. jeddahense* strain EE-R19 to Rapid Annotation using Subsystem Technology (RAST) server [11]. The number of rRNA and tRNA genes was further recognized by using Barrnap (version 0.9) [12] and Aragorn (version 1.2) [13], respectively.

After filtering and quality assessment, reads were then assembled into 59 contigs (> 500 bp), finally creating a genome with a total size of 3,562,974, having an average G + C content of 51.79%. The RAST server predicted 3972 protein-coding genes. Most of the annotated genes determined the synthesis of amino acids and derivatives (258), carbohydrates metabolism (253), protein metabolism (164), synthesis of cofactors, vitamins, prosthetic groups and pigments (122), and fatty acids, lipids and isoprenoids metabolism (32). Barrnap and Aragorn predicted 5 rRNA and 47 tRNA genes, respectively. The strain *C. jeddahense* strain EE-R19 has several genes involved in the biodegradation of carbohydrates and proteins, mixed acid and lactate fermentation, butanol biosynthesis, as well as in the metabolism of acetoin and butanediol. Moreover, several genes have been identified that are responsible for resistance to toxic compounds, including copper, cobalt, zinc, and cadmium. The genome data presented here should contribute to further research on this organism.

## Limitations

The exact genome length, synteny, number of rRNA genes, and repetitive elements cannot be certainly reported since the data obtained is based on the draft level genome sequence.

## Data Availability

The data described in this Data note can be freely and openly accessed at DDBJ/ENA/GenBank. Accession Numbers–https://www.ncbi.nlm.nih.gov/nuccore/JAAVNF000000000.1 (whole genome project) [14] and https://identifiers.org/ncbi/insdc:MT253587.1 (16S rRNA gene sequence) [15].
